# Impaired Release of Antimicrobial Peptides into Nasal Fluid of Hyper-IgE and CVID Patients

**DOI:** 10.1371/journal.pone.0029316

**Published:** 2011-12-27

**Authors:** Andreas Cederlund, Marie Olliver, Rokeya Sultana Rekha, Monica Lindh, Lennart Lindbom, Staffan Normark, Birgitta Henriques-Normark, Jan Andersson, Birgitta Agerberth, Peter Bergman

**Affiliations:** 1 Department of Medical Biochemistry and Biophysics, Karolinska Institutet, Stockholm, Sweden; 2 Department of Microbiology, Tumor and Cellbiology, Karolinska Institutet, Stockholm, Sweden; 3 International Centre for Diarrheal Diseases Research, Bangladesh, Dhaka, Bangladesh; 4 Department of Physiology and Pharmacology, Karolinska Institutet, Stockholm, Sweden; 5 Department of Medicine, Center for Infectious Medicine, Karolinska Institutet, Stockholm, Sweden; 6 Department of Laboratory Medicine, Division of Clinical Microbiology, Karolinska University Hospital, Huddinge, Sweden; McGill University, Canada

## Abstract

**Background:**

Patients with primary immunodeficiency (PID) often suffer from frequent respiratory tract infections. Despite standard treatment with IgG-substitution and antibiotics many patients do not improve significantly. Therefore, we hypothesized that additional immune deficits may be present among these patients.

**Objective:**

To investigate if PID patients exhibit impaired production of antimicrobial peptides (AMPs) in nasal fluid and a possible link between AMP-expression and Th17-cells.

**Methods:**

Nasal fluid, nasopharyngeal swabs and peripheral blood mononuclear cells (PBMCs) were collected from patients and healthy controls. AMP levels were measured in nasal fluid by Western blotting. Nasal swabs were cultured for bacteria. PBMCs were stimulated with antigen and the supernatants were assessed for IL-17A release by ELISA.

**Results:**

In healthy controls and most patients, AMP levels in nasal fluid were increased in response to pathogenic bacteria. However, this increase was absent in patients with common variable immunodeficiency (CVID) and Hyper-IgE syndrome (HIES), despite the presence of pathogenic bacteria. Furthermore, stimulation of PBMCs revealed that both HIES and CVID patients exhibited an impaired production of IL-17A.

**Conclusion:**

CVID and HIES patients appear to have a dysregulated AMP response to pathogenic bacteria in the upper respiratory tract, which could be linked to an aberrant Th17 cell response.

## Introduction

Diseases that are characterized by lack of antibodies (hypogammaglobulinemias, HGGs) include syndromes such as selective IgA-deficiency, IgG-deficiency and common variable immunodeficiency (CVID). Common symptoms include frequent bacterial respiratory tract infections (RTIs), fatigue as well as autoimmune manifestations [1]. Bacteria are the most common infectious agent, although viral, fungal and protozoal infections may also occur [2]–[5]. Standard treatment involves antibiotic therapy and patients with low levels of IgG and/or IgA are administered IgG preparations, which in most cases reduce symptoms and improve overall health [6]. Nevertheless, many patients still experience frequent infections. Hence, it is possible that immune deficits other than lack of immunoglobulins may be involved in some of these disorders. Such immune deficits could include two key components of the innate and adaptive immune system, namely antimicrobial peptides (AMPs) and Th17 cells.

AMPs are expressed by immune and epithelial cells and constitute an essential part of innate immunity [7]. They are released early during infection and have potent antimicrobial activities against a wide range of microbes. AMPs also induce chemotaxis of neutrophils and macrophages, which are rapidly recruited to the site of infection [8]. AMPs have been implicated in several human diseases, including psoriasis [9], [10], Crohn's disease [11], cystic fibrosis [12] and tuberculosis [13].

Th17 cells constitute a recently discovered subset of T helper cells characterized by expression of the transcription factor RORγt and secretion of IL-17 and IL-22 [14]. They are instrumental in mucosal immunity by orchestrating AMP expression in epithelial cells as well as by recruiting neutrophils to mucosal tissues [15]. Th17 cells are generally considered to be pro-inflammatory and have been shown to mediate autoimmunity in both rodents and humans [16]. Furthermore, studies using animal models have demonstrated a role for Th17 cells in mediating protection against several different bacteria and fungi, suggesting dual and context-dependent roles for Th17 cells [17], [18]. Recently, a number of human disorders characterized by infectious susceptibility and low numbers of Th17 cells have been described, including Hyper-IgE syndrome (HIES) [19] and chronic mucocutaneous candidiasis [20]. Special focus has been given to HIES, a PID characterized by frequent skin and RTIs together with distinct skeletal abnormalities and very high levels of serum-IgE. The HIES diagnosis is obtained by genetic analysis of the STAT3 gene [21].

Here we sought to investigate whether induction of AMPs and Th17 cell responses are impaired in PID patients with frequent RTIs. We collected nasopharyngeal (NPH) swabs, nasal fluid and peripheral blood mononuclear cells (PBMC) from patients with selective IgA-, or IgG-deficiencies, CVID, or with frequent RTIs without a defined diagnosis (Not Defined, ND group). In addition, two patients with HIES carrying mutations in the STAT3 gene were enrolled. NPH swabs were screened for bacterial growth while nasal fluid samples were investigated for AMP expression, IL-8 levels and induction of neutrophil chemotaxis. PBMCs were challenged with antigens and IL-17A was measured in the supernatant. Finally, a clinical scoring system was used to assess the clinical status of the patient at the time of inclusion.

## Methods

### Ethics statement

The study was approved by the Regional Ethical Committee in Stockholm, FE 289, Nobels väg 9, Karolinska Institutet, 171 77 Stockholm, Sweden (DNR 2007/751-31/1-4) and written informed consent was collected from all patients prior to inclusion and sample-collection.

### Patients and healthy controls

All patients were recruited at the Immune Deficiency Unit, Karolinska University Hospital, Huddinge. The diagnostic criteria were the following: **XLA (D80.0)**: a mutation in the btk-gene leading to a total lack of endogenous synthesis of immunoglobulins (n = 3). **CVID (D83.0)**: serum-IgG<3 g/l, serum-IgA<0.07 g/l and a lack of IgG response to pneumo-coccal polysaccharide vaccination (Pneumovax). No other diagnosis should explain the lack of IgG and IgA (n = 18). **IgG-subclass deficiency (D80.3)**: serum-IgG1<2.8 g/l; s-IgG2<1.15 g/l; s-IgG3<0.24 g/l (n = 55 for the whole group of subclass deficiencies) or total serum IgG<6.7 g/l (n = 9). **Selective IgA-deficiency (D80.2)**: serum-IgA<0.07 g/l and normal IgM- and IgG-levels (n = 16). All IgA-deficient patients included in the study exhibited at least four bacterial sinopulmonary infections per year. To obtain sufficient numbers for statistical calculations XLA, IgG-deficiency and selective IgA-deficiency were analyzed together in the results-section. **HIES (D82.4)**: mutation analysis of the STAT3 gene was performed by the Grimbacher lab (UCL School of Life and Medical Sciences, London), (n = 2). Patient HIES1 had a heterozygous mutation (Y657C) and serum IgE of 7600 units. Patient HIES2 had a heterozygous mutation (R382L) and serum IgE of 23000 units. **Patients with increased numbers of respiratory tract infections (Not Defined, ND-group, D89.9)**: at least 4 RTIs per year during the last 2 years that required antibiotic treatment and/or recurrent episodes of virus-like symptoms with muscle pain, malaise and fever during at least 3 months per year during the last 2 years, with significant negative impact on quality of life (n = 30). 26 healthy controls were recruited as well as 5 control individuals with ongoing infections and symptoms from the respiratory tract, such as runny nose, cough and malaise.

### Clinical scoring system

On the day of study inclusion, the patients were investigated by a physician who recorded the following symptoms: symptoms from ears, sinuses, nose, bronchitis, pneumonia, cough, runny nose, sore throat, fever, fatigue or malaise. Each symptom was given 1 point and the scoring range was between 0 points (no symptoms) and 11 points (maximum score).

### Sampling of nasal fluid, NPH swabs

A nasopharyngeal swab was taken from one nostril and sent to the Clinical Microbiology Laboratory at Karolinska University Hospital, Huddinge for bacterial culture. The bacterial content was evaluated as either “no growth of bacteria”, “normal flora” (typical findings include alpha haemolytic streptococci, *corynebacteria* spp, commensal *Neisseria* spp. and other non-pathogenic strains) or “pathogenic growth” (defined here as *H. influenzae*, *S. aureus*, *S. pneumoniae*, *M. catharralis* and *enterobacteriacae* spp). Subsequently, nasal fluid was collected through a thin plastic tube carefully inserted into the back of the nose using the other nostril as entry port (10–12 cm from the nostril meatus). 5–10 ml of saline was administered prior to sampling in order to make the epithelial lining moist and to dissolve mucus depositions. A gentle vacuum was applied and 3–5 ml nasal fluid was collected and stored at −20°C.

### Extraction of peptides and proteins from nasal fluid

Nasal fluid (3–5 ml) was extracted in an equal volume (1∶1) of 60% acetonitrile (AcN) in 1% trifluoroacetic acid (TFA) over night at 4°C. The extract was centrifuged at 3500 g and the supernatant was lyophilized. The lyophilized extract was resuspended in 0.1% TFA and enriched for polypeptides using solid phase extraction as described in [22]. The lyophilized polypeptide extract was reconstituted in 0.1% TFA to a concentration of 5 µg/µl as determined spectrophotometrically using a Nanodrop-system (Thermo Scientific, Wilmington, U.S.).

### Lithium dodecyl sulfate polyacrylamide gel electrophoresis (LDS-PAGE) and Western blotting

The concentrated and lyophilized extract (25 µg) was dissolved in lithium dodecyl sulphate (LDS) sample buffer, 50 mM Dithiothreitol (DTT) (Sigma-Aldrich, St. Louis, Missouri, USA) and incubated at 70°C for 10 min. The samples were then separated using LDS-PAGE and blotted onto PVDF membranes as described in [22]. Antibodies used were a LL-37 monoclonal [23] and a HNP1–3 goat polyclonal (sc-22916, Santa Cruz, Santa Cruz, Calif., USA). Proteins and peptides were visualized on chemiluminescence film with ECL plus Western blot detection system (GE Healthcare, Buckinghamshire, United Kingdom).

Concentrations of LL-37 and HNP1–3 in nasal fluid were determined by densitometry using the software ImageJ (http://rsbweb.nih.gov/ij/). The intensity of each band was normalized to an external standard on each membrane and the total amount of LL-37 and HNP1–3 was determined by multiplying the densitometric result (ng peptide/µg extract) with the total amount of polypeptide-extract (µg). Thus, the values represent the total amount of LL-37 and HNP1–3 from each nasal fluid sample.

### Enzyme-linked immunosorbent assay (ELISA) of IL-8 and HBD-2

The concentrated extracts were diluted 1∶500 and 1∶3000 in sample diluent for the HBD-2 (Alpha diagnostic, Texas, USA) and OptEIA IL-8 (BD Biosciences, New Jersey, USA) ELISA respectively. ELISAs were performed according to the manufacturers' instructions and samples were run in triplicate in two independent experiments.

### Isolation of polymorphonuclear leukocytes (PMNs)

Human PMNs were isolated from freshly prepared buffy coats from healthy donors (Karolinska Hospital Blood Bank, Stockholm, Sweden) in accordance with [22]. PMNs were suspended to 10^6^ cells/mL in HBSS (Invitrogen, Carlsbad, CA, USA). The viability of PMNs was determined with Trypan blue (Invitrogen, Carlsbad, CA, USA) staining.

### Neutrophil chemotaxis

PMN chemotaxis was measured in a modified Boyden chamber assay. Briefly, 10^5^ human PMNs in HBSS (Invitrogen, Carlsbad, CA, USA) were added to the upper chamber of a 3 µm pore polycarbonate Transwell culture insert plate (Corning, NY, USA) and incubated at 37°C for 2 hours in the absence or presence of 10 µg concentrated nasal fluid extract in the lower chamber. IL-8 (10 nM) (BD Pharmingen, San Diego, CA, USA) was used as a positive control for migration across the filter. The filters had previously been blocked with 0.1% BSA in PBS for 30 min. To efficiently remove all transmigrated cells from the lower chamber, a final concentration of 10 mM EDTA was added to each well and cells were resuspended. Thereafter, the cell number was determined in a FACSCalibur (Beckton Dickinson, San Jose, CA, USA) by gating on the forward and side scatter characteristics.

### Stimulation of PBMCs and IL-17A ELISA

PBMCs were isolated from whole blood using Lymphoprep, according to the manufacturer's instructions. Cryopreserved PBMCs were quickly thawed at 37°C, washed twice and resuspended in IMDM (Sigma-Aldrich), supplemented with 10% heat-inactivated FCS and 2 mM L-glutamine (Invitrogen). PBMCs (5×10^5^ cells per well) were stimulated with 20 µg/ml *Candida albicans* antigen (Greer Labs, Lenoir, NC, USA) or 100 ng/ml Staphylococcal enterotoxin B (SEB) (Sigma-Aldrich) in 96-well flat bottom plates. Experiments were performed in triplicates and stimulated cells were incubated at 37°C in a humidified atmosphere with 5% CO_2_. Supernatants were collected after 5 days of stimulation and stored at −20°C until assayed for IL-17A using a Ready-SET-Go! ELISA kit (eBioscience, San Diego, CA, USA), performed according to the manufacturer's instructions.

### Statistical methods

Data was processed using the softwares Microsoft Excel and Graph Pad Prism. Normal distribution tests were performed and most data sets were found to be non-normally distributed. Thus, non-parametric tests (Mann Whitney U-test) were used where applicable. Two-sided tests were used for all analyses and p-values<0.05 were considered significant.

## Results

### Clinical characteristics of patients and healthy controls

A total of 155 samples were collected from patients with various PID diagnoses and healthy controls (Table 1). All patients submitted a clinical scoring card (see Methods for details) before sample collection. No significant differences were found in clinical score between the different groups (p = 0.23, Kruskal-Wallis test). Healthy individuals with an ongoing infection exhibited a clinical score of 4.4, however, a positive bacterial culture was only detected in 40% of these patients, indicating that the sampling method used has a low specificity [24] or that also viral infections could be involved [2]. A total of 23% (range 0–50%) of all individuals in the study exhibited growth of a primary pathogen in nasal swabs, including the healthy controls with a clinical score of zero. There was no correlation between the clinical score and the presence of potentially pathogenic bacteria in the NPH culture. Out of all patients, 51% were treated with IgG-substitution, with the highest treatment fraction in XLA, HIES- and CVID patients (Table 1), which is in line with current guidelines [25]. In fact, XLA- and CVID-patients are subjected to lifelong IgG replacement therapy based on the fact that they have an impaired endogenous production of immunoglobulins [26]. Also patients with IgG subclass deficiencies have been shown to benefit from IgG replacement therapy [27]. Moreover, in our centre we evaluate the efficacy of IgG substitution to selected patients with frequent bacterial infections but with normal IgG-levels [28].

**Table 1:** Clinical characteristics of patients and controls.

Diagnosis	No of samples	% P growth (*n* samples)	Clin Score (average)	% Ig-subst (*n* samples)
**Healthy Controls**	**26**	**12 (3)**	**0.0**	**0 (0)**
**Infected Controls**	**5**	**40 (2)**	**4.4**	**0 (0)**
**Not Defined Immunodeficiency (ND) D89.9**	**30**	**33 (10)**	**2.5**	**47 (14)**
**IgA/IgG group (IgA, IgG, XLA)**	**74**	**19 (14)**	**3.1**	**64 (47)**
*-Selective IgA-deficiency D80.2*	*16*	*31 (5)*	*2.6*	*38 (6)*
*-IgG subclass deficiency D80.3*	*55*	*15 (8)*	*3.2*	*69 (38)*
*-XLA D80.0*	*3*	*33 (1)*	*3.7*	*100 (3)*
**HIES D82.4**	**2**	**50 (1)**	**5.5**	**100 (2)**
**CVID D83.0**	**18**	**28 (5)**	**2.6**	**89 (16)**
Total	**155**	**23 (35)**	**3.0**	**51 (79)**

% P growth = Percent of samples with growth of bacterial pathogens in the nasopharynx. The values in brackets designate the number of samples with growth of a pathogen.

Clin Score = clinical score from 0 (no symptoms)−to 11 (for details see the Methods section).

% Ig-subst = Percent of patients that receive immunoglobulin treatment.

### The bacterial composition in the nasopharynx dictates levels of AMPs in nasal fluid

**Figure 1:**
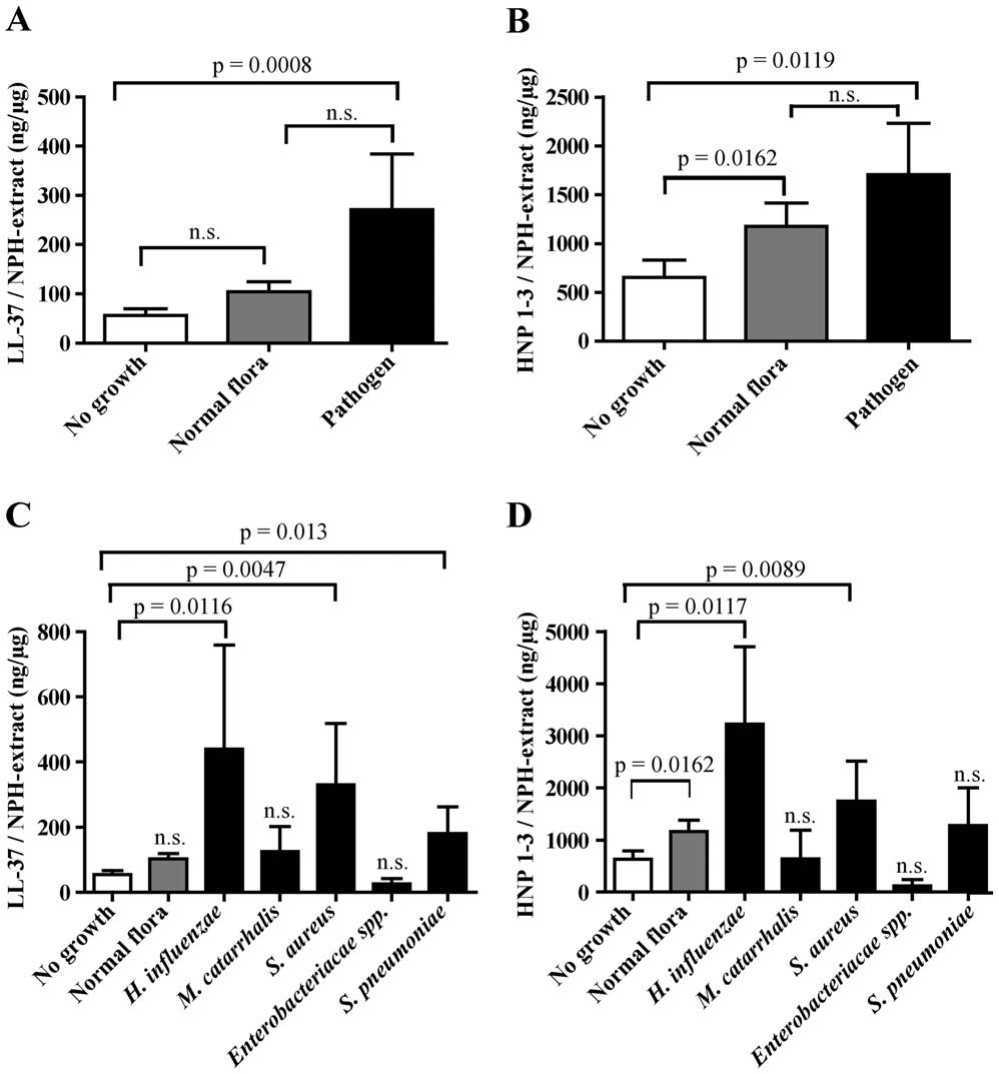
Bacterial composition and levels of AMPs in nasal fluid. AMP levels in nasal fluid samples were determined by Western blotting and subsequent densitometric measurements and the results were correlated to bacterial growth in the nasopharynx. The levels of LL-37 (A and C) and HNP1–3 (B and D) are expressed as nanogram peptide/total nasal fluid extract (µg). The group that exhibited growth of primary pathogens was further subdivided according to bacterial species (C and D). The number of samples included (no growth, normal flora, and pathogen respectively) are n = 44, 43, and 30 (A), n = 39, 34, and 28 (B). The number of samples included (no growth, normal flora, *H. influenzae*, *M. catarrhalis*, *S. aureus*, *Enterobacteriacae*, *S. pneumoniae* respectively) are n = 44, 43, 8, 3, 11, 3, 5 (C), n = 39, 34, 7, 3, 11, 2, 5 (D). Statistical analyses were performed with Mann Whitney U-test and the standard error of the mean (SEM) was used to estimate sample variation.

AMP levels in nasal fluid were determined by Western blot analysis and subsequent densitometric measurements. Interestingly, patients and healthy controls colonized with a primary pathogen, here defined as *Moraxella catarrhalis, Staphylococcus aureus, Haemophilus influenzae, Streptococcus pneumoniae* and *Enterobacteriaceae spp.*, exhibited significantly increased levels of AMPs (LL-37, Fig. 1A; HNP1–3, Fig. 1B) compared to individuals with a negative bacterial culture. Furthermore, LL-37 levels in nasal fluid from individuals with normal flora were not significantly elevated compared to individuals where no bacteria were detected (Fig. 1A). However, HNP1–3 levels were significantly increased if normal flora was detected compared to samples from individuals with no growth (Fig. 1B). Bacteria associated with the highest AMP concentrations were *H. influenzae*, *S. aureus* and *S. pneumoniae* (Fig. 1C and D, respectively). AMP levels associated with growth of *Enterobacteriacae spp.* and *M. catarrhalis* were not significantly elevated (Fig. 1C and D).

### Expression of AMPs in nasal fluid is the result of a dynamic response to bacteria

**Figure 2:**
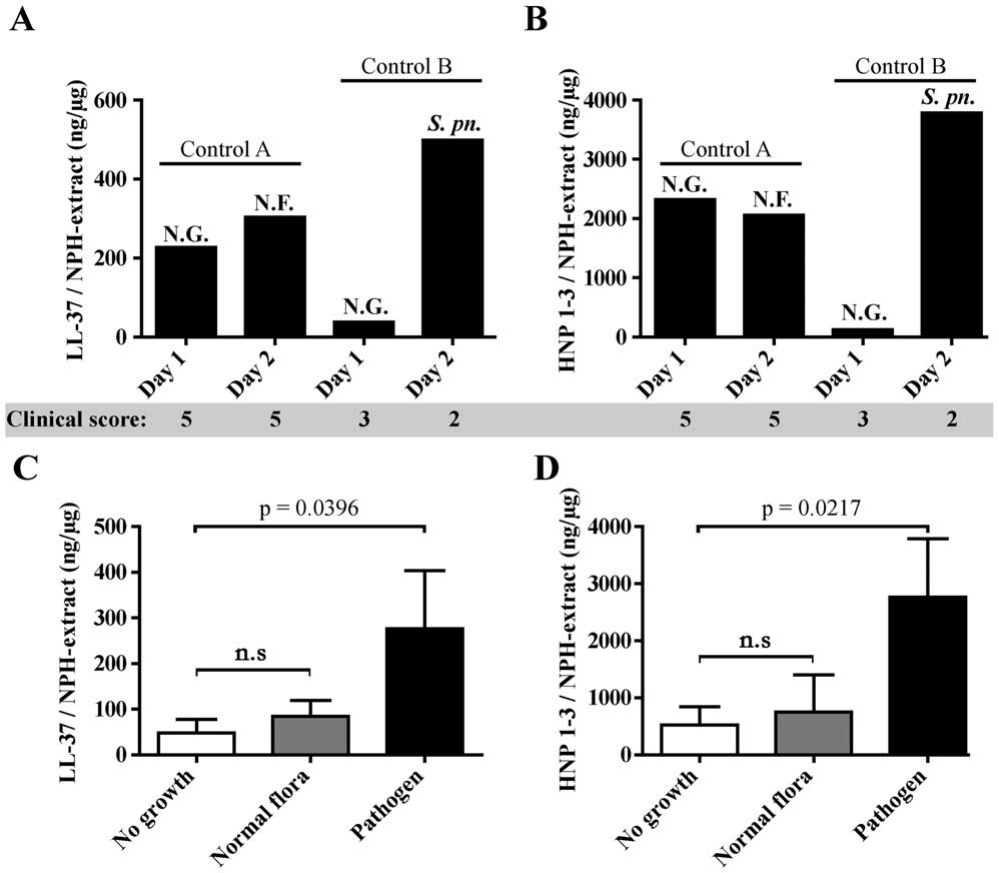
AMP response in nasal fluid of healthy controls. Two previously healthy controls (A, female, age 36; B, male, age 38) were sampled for nasal fluid on two consecutive days and AMP levels were determined. The total levels of LL-37 (A) and HNP1–3 (B) are expressed as nanogram peptide/total nasal fluid extract (µg). The clinical score prior to sampling was also measured (A and B). The whole group of controls (both healthy and infected controls as determined by the clinical score) was analyzed for levels of LL-37 (n = 18) (C) and HNP1–3 (n = 12) (D). The number of samples (no growth, normal flora, pathogen, respectively) are n = 7, 8, and 3 (C), n = 7, 3, and 2 (D). Error bars depict the SEM of the samples (C and D).

AMP levels were measured in nasal fluid samples from 26 healthy individuals and from 5 previously healthy individuals which were defined by the clinical score as having on-going infections, (See Materials & Methods for details and Table 1). For two healthy infected individuals (Control A, female, age 36 and Control B, male, age 38), samples were obtained on two consecutive days (Fig. 2A and B). Nasal swabs were cultured for the presence of bacteria and levels of LL-37 and HNP1–3 were determined. Control A had a negative bacterial culture day 1 (no growth, NG) and growth of normal flora (NF) day 2. This change in bacterial composition had only a moderate impact on LL-37 and HNP1–3, which is in line with previous results (Fig. 1A and B). Control B had no growth of bacteria day 1 (NG) while the primary pathogen *S. pneumoniae* (*S. pn*) was isolated day 2. In accordance with results presented in Figure 1A and B, this individual exhibited an increased production of both LL-37 and HNP1–3 in response to colonization with a pathogen. Notably, control A with a clinical score of 5 exhibited high levels of both LL-37 and HNP1-, despite the absence of pathogenic bacteria in the nasal swab. Thus, a possible explanation for this finding is that a viral infection caused control A's symptoms through a local inflammation and subsequent AMP-release. In contrast, control B had an initial score of 3, which decreased to 2 on the second day of sampling, despite being culture-positive for *S. pneumoniae*. Therefore the rapid AMP release in control B is most likely explained by a transient pneumococcal colonization rather than a symptomatic infection. Again, these data imply that the regulation of AMPs occurs independently of clinical symptoms. When the total group of healthy and infected controls was evaluated, a significant induction of both LL-37 (n = 18, p = 0.0396, Fig. 2C) and HNP1–3 (n = 12, p = 0.0217, Fig. 2D) in response to primary pathogens was detected, supporting the results presented in Figure 2A and B. A nasal swab with growth of “normal flora” was not associated with a significant induction of LL-37 or HNP1–3 (Figure 2C and D) in these healthy controls.

### Release of AMPs in response to pathogenic bacteria is significantly impaired in CVID and HIES patients

**Figure 3:**
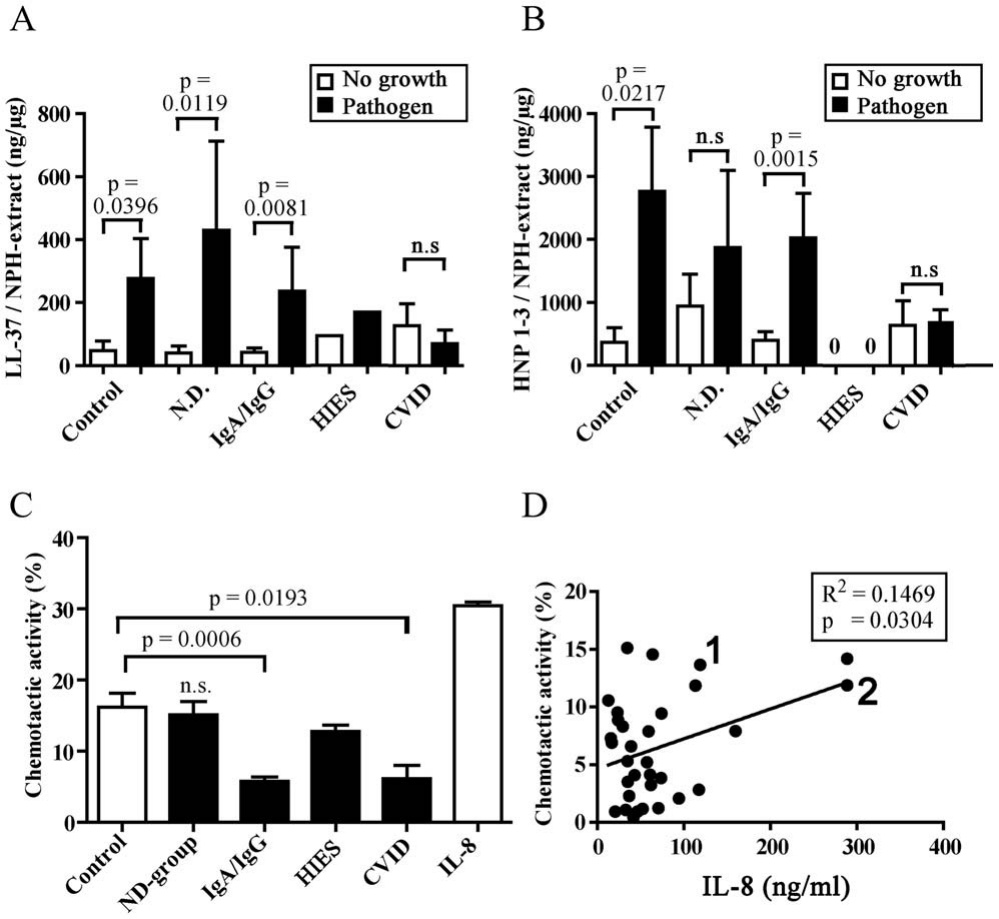
AMP response and chemotaxis in relation to diagnosis. AMP levels were studied according to diagnosis and presence of bacterial pathogens. “No growth” designates negative bacterial cultures and “Pathogen” designates growth of a primary bacterial pathogen (A and B). The number of samples included (control, ND, IgA/IgG, HIES, and CVID respectively with and without growth) are n = 7, 3, 11, 9, 27, 14, 1,1, 6, 4 (A), n = 7, 2, 11, 10, 28, 13, 1,1, 6, 4 (B). The chemotactic capacity was determined for samples from each diagnostic group and is expressed as percent migrated neutrophils (C). The numbers of samples in C are: n = 5, 10, 44, 2, 10, 7. Error bars depict the SEM of a sample. Levels of IL-8 in nasal fluid (n = 32) were plotted against the chemotactic index and a weak positive correlation was found (p = 0.03, R^2^ = 0.14) (D). HIES-patients 1 and 2 are marked “1” and “2”. Statistical analyses in figure A, B and C were performed with Mann Whitney U-test and for figure D a correlation test was used.

Next, the AMP response in the immunodeficient patient groups with regard to bacterial pathogens in the nasopharynx was studied (Fig. 3A and B). A significant increase in LL-37 levels was observed in controls colonized by pathogens (same data as in fig. 2C). This increase in AMP-levels was also true for patients with no determined immune disorder (N.D. group) and for the IgA/IgG group (Fig. 3A). XLA-patients (n = 3) had an AMP-response to pathogenic bacteria in the same range as healthy controls (data not shown) and were presented together with the IgA/IgG-group in figure 3A and B. HNP1–3 levels in the control group and in IgA/IgG patients were also significantly increased when pathogens were present, and while a similar trend was noted for the N.D. group, it did not reach statistical significance (Fig. 3B). Notably, there was no increase in neither LL-37 nor HNP1–3 in the CVID or HIES patients, despite bacterial growth (Fig. 3A and B, table 2). In fact, both HIES patients completely lacked HNP1–3 in the nasal fluid (Fig. 3B).

**Table 2:** Summary of results obtained in the study.

	AMP induction			
	LL-37	HNP1–3	Chemotaxis	PBMC (IL-17A)
**Controls**	+	+	+	+
**ND group**	+	+	+	+
**IgA/IgG**	+	+	−	+
**HIES**	−	−	+	−
**CVID**	−	−	−	+/−

**Figure 4:**
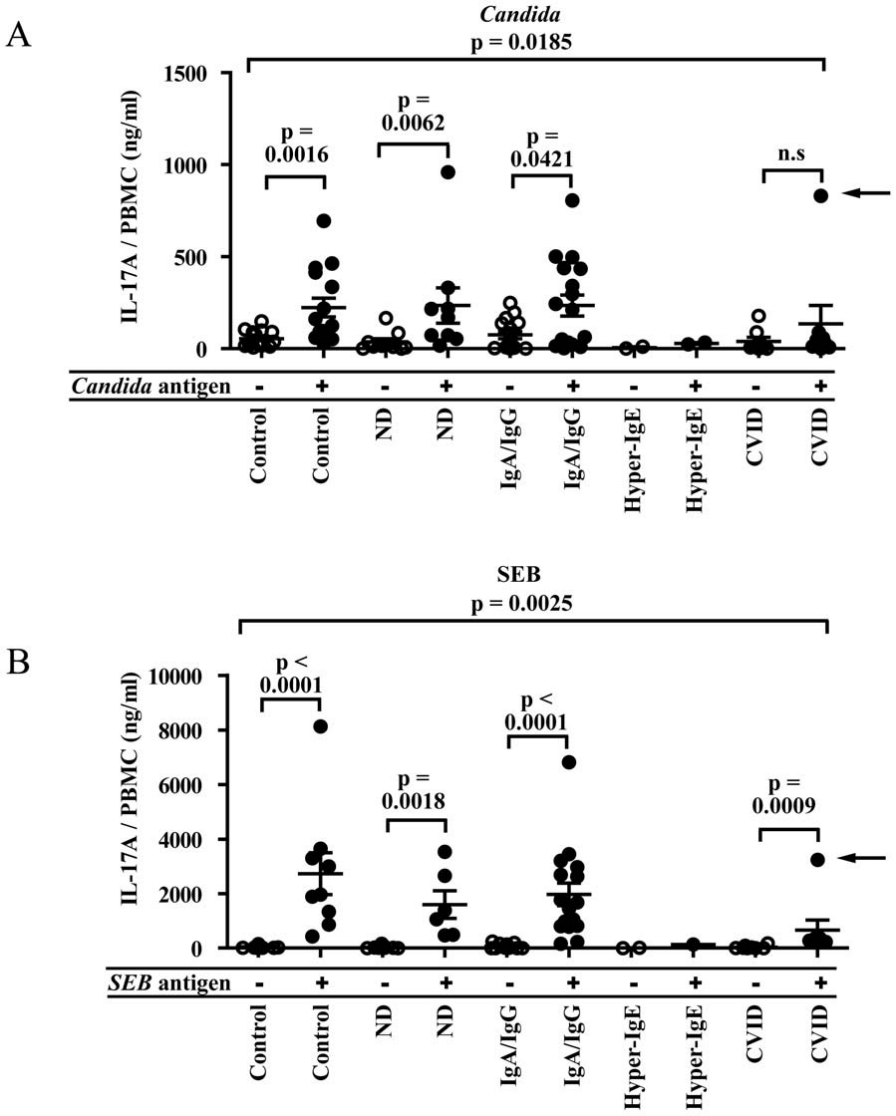
Stimulation of PBMCs with Candida antigen and SEB. PBMCs isolated from healthy controls and patients were stimulated with *Candida* antigen (A) and SEB (B), respectively. After 5 days, IL-17A levels were measured in the supernatants with ELISA. U; unstimulated, C; stimulated with *Candida* antigen, and S; stimulated with SEB antigen. The number of samples are n = 15, 15, 9, 9, 17, 17, 2, 2, 8, 8 (A), n = 15, 9, 9, 6, 17, 16, 2, 1, 8, 8 (B). Bars represent the mean value. Mann Whitney U-test was used in A and B.

A summary of the responses for the different patient groups. A “+”-sign denotes a significant response with regards to AMP-induction, chemotaxis or IL17-A release from PBMC-cultures, respectively. A “−”-sign represent a non-significant response. “+/−” means that CVID-patients respond with a significant increase in IL17-A after stimulation with SEB, but not candida. Moreover, CVID-patients responded significantly less than healthy controls (see also fig. 4A and B).

### Chemotactic activity of nasal fluid towards healthy control neutrophils

We then wanted to investigate the reason for the dysregulated release of AMPs into nasal fluid of HIES and CVID patients. Nasal fluid derived from patients and controls was investigated for neutrophil chemotaxis using neutrophils isolated from healthy control individuals (Fig. 3C). These results revealed that nasal fluid collected from the N.D group exhibited a chemotactic activity that was indistinguishable from control individuals (Fig. 3C). In contrast, patients with IgA/IgG-deficiency and CVID showed significantly reduced chemotactic activity compared to controls (Fig. 3C). Unexpectedly, the two HIES-patients exhibited the same chemotactic activity as control individuals (table 2).

To further dissect the chemotactic activity of nasal fluid, the concentration of the main chemoattractant for neutrophils, IL-8, was determined by ELISA. There was a weak - although statistically significant - correlation between the chemotactic activity and IL-8 levels in nasal fluid, indicating a contribution of this cytokine to the observed chemotaxis (p = 0.0304 and R^2^ = 0.1469) (Fig. 3D). Notably, HIES patients 1 and 2 appeared in the upper right quadrant of the figure, indicating high chemotactic activity as well as high IL-8 levels (Fig. 3D, marked with “1” and “2”, respectively).

### PBMCs from CVID and HIES patients release low levels of IL-17A

To investigate if AMP expression in nasal fluid could be correlated with the IL-17A response from circulating immune cells, we measured release of IL-17A in stimulated PBMCs from patients and healthy controls (Fig. 4). PBMCs were isolated from whole blood and stimulated for 5 days with *Candida* antigen (Fig. 4A) or Staphylococcal Enterotoxin B (SEB) (Fig. 4B). IL-17A production in PBMCs isolated from healthy controls, IgA/IgG patients as well as from patients of the N.D. group, exhibited a similar and robust increase in IL-17A after stimulation. As expected, samples from HIES patients contained very low levels of IL-17A. PBMCs from CVID patients did also respond to antigen stimulation with release of IL-17A (n.s. for *Candida* antigen and p = 0.0009 for SEB) (Fig. 4A and B). Interestingly, this release was significantly lower than the release observed for cells obtained from healthy controls (p = 0.0185 for *Candida* and p = 0.0025 for SEB, table 2). Thus, these results imply that CVID-patients – similarly to HIES patients – have an impaired release of IL-17A from SEB- and Candida stimulated PBMCs. If this problem stems from aberrant recognition of antigen or intrinsic abnormalities in IL-17A production, remains to be elucidated.

The major findings of this report are summarized in table 2. A (+)-sign in columns “LL-37” and/or “HNP1–3” represent an increased release of LL-37 and/or HNP1–3 in response to pathogenic bacteria comparable to that of the control group, whereas those denoted by a (−)-sign exhibit an impaired release. Patient groups marked with (+) in the “Chemotaxis” column showed a chemotactic activity against healthy PMNs comparable to the control group and a (−)-sign denotes impaired or lack of chemotactic activity. A (+)-sign in the “PBMC” column refers to a capacity of the PBMCs to release IL-17A in response to Candida or SEB antigens, a (−)-sign is indicative of an impaired IL-17A release, whereas +/− is indicative of statistically significant response to SEB antigen, but not to Candida antigen (CVID-patients).

## Discussion

**Figure 5:**
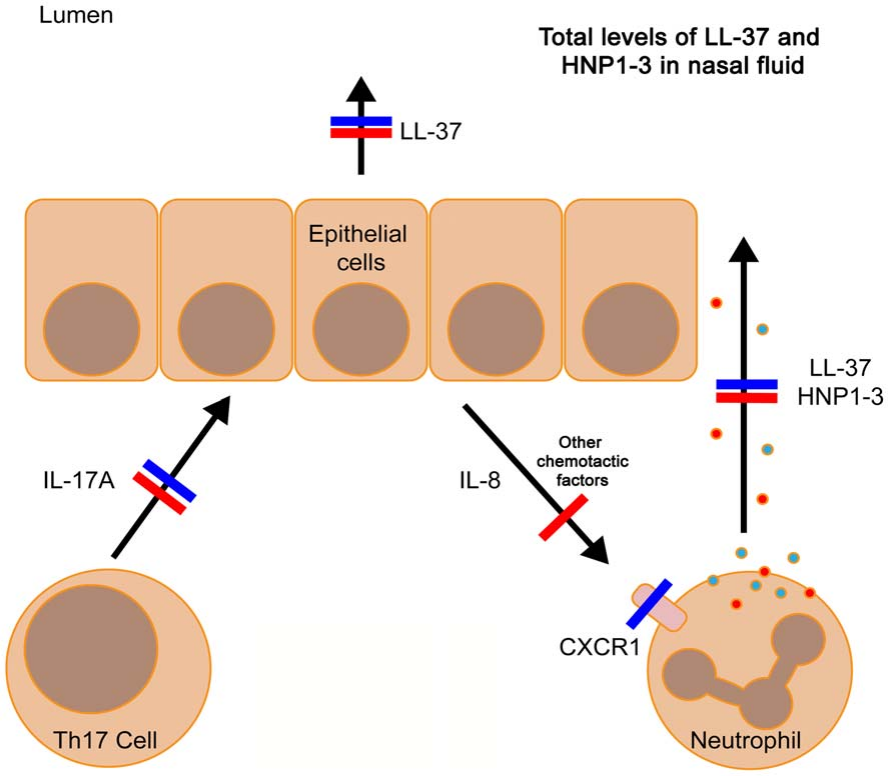
Impaired release of AMPs into nasal fluid of HIES- and CVID patients – A proposed model. HIES patients (blue lines) exhibit an impaired production of IL-17A and their nasal fluid contain very low levels of LL-37 and HNP1–3 despite normal chemotactic activity and IL-8 levels. This may be explained by defects in expression of the IL-8 receptor CXCR1, resulting in non-responding neutrophils. CVID patients (red lines) also exhibited a dysregulated release of AMPs, which may be explained by the low chemotactic activity of nasal fluid as well as an impaired Th17 response. However, CVID patients have, in contrast to HIES patients, normally responding neutrophils, but this may not be sufficient, since the chemotactic signal is too weak.

In this study we investigated if altered levels of AMPs in nasal fluid may explain why PID patients frequently acquire RTIs. A key finding was that most patients and healthy controls responded to pathogenic bacteria with an increased AMP expression in nasal fluid. However, this response was impaired in CVID and HIES patients, which may be due to a low response from IL-17A producing cells, including CD4+ Th17-cells, NK-cells or monocytes [29]. However, IL-17A has been described as the “signature cytokine” for Th17-cells and is – together with IL-22 – implicated in mucosal immunity against bacterial and fungal infections [15]. The role of IL-17A and IL-22 producing immune cells in mucosal immunity is based on the assumption that these cells migrate to the mucosa during infection where they orchestrate local immune responses, such as neutrophil chemotaxis (via IL-8) or induction of AMP synthesis (direct effects of IL-17A and IL-22 on epithelial cells) [15]. Our data indicate that HIES and CVID patients exhibit a similar impaired release of AMPs into nasal fluid, but with separate immunological pathways involved (summarized in table 2 and figure 5).

The two HIES patients with STAT3 gene mutations responded with very low levels of IL-17A in antigen-stimulated PBMCs, which is in line with a previous study [19]. Nasal fluid from these two patients contained low levels of LL-37 and completely lacked HNP1–3. LL-37 is expressed by both immune and epithelial cells, whereas HNP1–3 is mainly present in neutrophils. Therefore, low or absent HNP1–3 in nasal fluid indicate an impaired recruitment of neutrophils to the nasal compartment. Indeed, HIES patients often suffer from “cold abscesses”; bacterial skin infection without neutrophil infiltration and classical signs of inflammation [21]. In chemotaxis experiments, nasal fluid from the two HIES patients induced normal chemotaxis in neutrophils derived from healthy individuals. Indeed, IL-8 levels in nasal fluid from these two HIES-patients were similar to controls (Figure 3D, numbers “1” and “2”), despite an almost complete lack of IL-17A production in PBMCs. This was unexpected but suggests that the low AMP levels in nasal fluid of HIES patients is not caused by a lack of chemotactic signals, but rather that neutrophils derived from HIES patients have impaired chemotaxis. In fact, it has been known since 1983 that neutrophils from HIES patients (Job's syndrome) do not respond properly to chemotactic signals such as complement (C5a) [30]. Moreover, deletion of the STAT3 gene in mice resulted in impaired neutrophil chemotaxis [31] and neutrophils from HIES patients display low levels of CXCR1, a receptor for IL-8 [32]. Thus, it is possible that the frequent mucosal infections among HIES patients is related to an impaired STAT3 mediated neutrophil migration, rather than impaired Th17 responses (Table 2 and Fig. 5).

In contrast, CVID patients were reported to have normally migrating neutrophils [30]. Nevertheless, we detected aberrant AMP responses to pathogenic bacteria in these patients. This could be explained by the significantly decreased chemotactic activity of nasal fluid from CVID patients (Fig. 3C). In addition, PBMCs derived from CVID patients responded with a weaker IL-17A response to SEB and *Candida* antigen, compared to control PBMCs (Fig. 4A and B). Thus, these data are in line with the current hypothesis that lack of IL-17A leads to a lower expression of mucosal AMPs, possibly caused by an aberrant chemotactic signal (Table 2 and Fig. 5). A recent paper from Barbosa et al, describes in detail that both CVID-patients and agammaglobulinemic patients (XLA-patients) exhibit Th17-cell disturbances, suggesting that B-cells are important for development of functional Th17-cells [33]. These data are in line with our results on CVID-patients but does not explain the mechanisms involved. Unfortunately, the low number of XLA-patients in our study precludes any conclusions regarding the role of Th17-cells in this disease. One possible explanation to the low IL-17A release from CVID-patients' PBMCs is that it reflects a general T-cell defect among CVID-patients, rather than a specific Th17-cell problem. Previously, CVID patients were shown to have lower numbers of CD4+ T cells, from which Th17 cells differentiate [34], [35]. This is in contrast to HIES-patients where a defined mutation in STAT3 is the underlying cause of the lack of Th17-cells, without general T-cell abnormalities [19]. SEB and candida induce Th17-cell differentiation by distinct pathways; SEB is a general T-cell activator by virtue of its superantigenic properties [36] whereas candida activates antigen presenting cells (APCs) via dectin-1 ligation [37]. Notably, SEB stimulation – but not candida stimulation – resulted in a small but statistically significant increase in IL-17A release from PBMCs of CVID-patients (figure 4A and B). In fact, it has been shown that CVID-patients have low numbers of dendritic cell subsets in peripheral blood [38]. Thus, it is possible that CVID-patients may have deficiencies both in the T- and dendritic cell compartments, although this speculation needs further work to become established. Interestingly, the CVID group contained one outlier with high IL-17A production. This patient was originally diagnosed with selective IgA-deficiency and has gradually declined in IgG-levels and was given the CVID diagnosis 2 years ago, which could explain the relatively high IL-17A levels (Fig. 4A and B, arrows).

We believe that the results presented here give further insight into the complex regulation of AMP production at mucosal surfaces of the respiratory tract in general and among PID patients in particular. The data presented here could also serve as a starting point for future studies, since the detailed mechanism for impaired IL-17A release among CVID patients merits further investigations. Finally, our data show that certain primary immune disorders, such as CVID and HIES are associated with mucosal dysregulation of AMP release, which provide exciting new avenues for clinical trials with AMP-inducing agents, such as vitamin D.
